# Impact of repeated percutaneous coronary intervention on long-term survival after subsequent coronary artery bypass surgery

**DOI:** 10.1186/1749-8090-6-107

**Published:** 2011-09-10

**Authors:** Genichi Sakaguchi, Takeshi Shimamoto, Tatsuhiko Komiya

**Affiliations:** 1 Department of Cardiovascular Surgery, Kurashiki Central Hospital, 1-1-1 Miwa, Kurashiki City, Japan

**Keywords:** coronary artery bypass grafting, coronary stent, prognosis

## Abstract

**(Background):**

In the current stent era, aggressive repeated percutaneous coronary intervention (PCI) has become more common. The aim of this study was to investigate the impact of previous repeated PCI on the subsequent coronary artery bypass grafting (CABG).

**(Methods):**

Between January 1990 and January 2008, a total of 894 patients underwent first-time isolated elective CABG. Among the 894 patients, 515 patients had had no PCI (group A), 179 patients had had single PCI (Group B), and 200 patients had had multiple PCI (2-15 times, mean 3.6 ± 2.3 times) (group C) before CABG. These groups were compared in terms of early and late clinical results.

**(Results):**

Preoperative left ventricular ejection fraction was significantly higher in group A (group A;58 ± 13%, group B;54 ± 12%, and group C;54 ± 12%). Number of bypass grafts was significantly smaller in group C (A:3.3 ± 1.0, B 3.4 ± 0.9, C 3.1 ± 1.0). Although there was no statistically significant difference among the groups, in-hospital mortality in group C was higher than that in group A and B (A:1.6%, B:1.1%, C:3.5%, p = 0.16). Survival analysis by Kaplan-Meier method (mean follow-up: 58 ± 43 methods) revealed that freedom from all-cause death and cardiac death was significantly lower in group C in comparison with group A. Freedom from cardiac event was significantly higher in group C than that in group A. Multivariate analysis identified a number of previous PCI as an independent risk factor for cardiac death.

**(Conclusions):**

Repeated PCI increased risk for long-term prognosis of subsequent CABG.

## Background

Although clinical trials comparing PCI with percutaneous coronary intervention (PCI) with coronary artery bypass grafting (CABG) in patients with multivessel coronary artery disease showed significant advantages with CABG in terms of the rate of repeat revascularization, major adverse cardiac event [1], and long-term survival [2,3] and the new ESC/EACTS guidelines on myocardial revascularization recommended CABG as the treatment of choice for patients with severe coronary artery disease [4], PCI has been increasingly used to treat complex coronary artery disease which had been thought to be a candidate for CABG as an initial treatment and aggressive repeated PCI with multiple stenting has been becoming more common in the "stent era". Consequently, CABG is reserved for patients who are not candidates for further PCI. Previous repeated PCI was reported to be a risk for perioperative mortality and morbidity in CABG [5-8], however, these studies have been limited to early outcomes and the impact of previous repeated PCI on mid-term outcomes of subsequent CABG is unclear. In the present study, we compared mid-term outcomes of patients who had CABG without previous PCI with those who had CABG with previous repeated PCI.

## Patients and Methods

The Institutional Review Board of Kurashiki Central Hospital approved this study, and waived the individual consent because this study was retrospective. Between January 1990 and January 2008, a total of 894 patients underwent first-time isolated elective CABG at Kurashiki Central Hospital. These patients were divided into 3 groups, according to whether they had no previous PCI (group A), a single previous PCI (group B), or multiple repeated previous PCI (group C) before CABG. Early and late clinical results were compared among the three groups. Cardiac death was defined as any cardiac-related, sudden, or unknown death. Cardiac event was defined as cardiac death, acute myocardial infarction, PCI, re-CABG, and congestive heart failure requiring hospitalization.

We examined the patients at our outpatient clinic or contacted the patients for follow-up. Follow-up was obtained on 93% of patients and the mean length of follow-up was 58 ± 43 months.

Continuous variables were presented as means with standard deviations (SD). Comparison of the clinical characteristics was performed by the chi-square analysis for categorical variables and by Student *t *test or ANOVA for continuous variables. Cumulative probability of survival was estimated with the Kaplan-Meier method and compared among the groups by using a log-rank test. Cox proportional-hazards regression models were used to determine the independent risk factors for death and cardiac events. Clinical variables with a value of p < 0.1 were incorporated into the multivariate models. Differences were considered significant at the level of p < 0.05. Data analysis was performed with StatView for Windows version 5.0 (SAS Institute Inc, Cary, NC).

## Results and discussion

### Results

**Figure 1:**
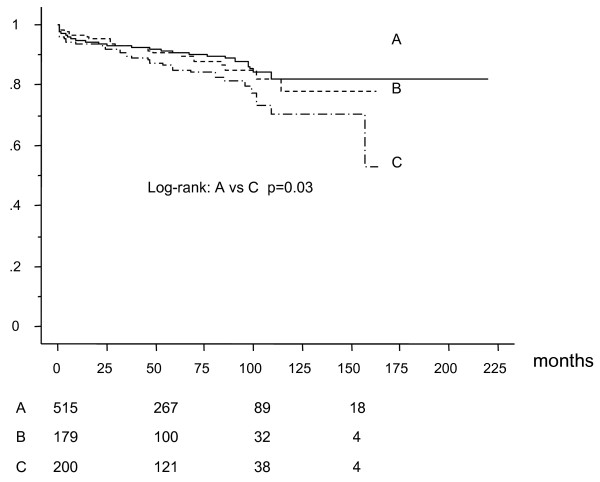
**Survival curve**.

**Figure 2:**
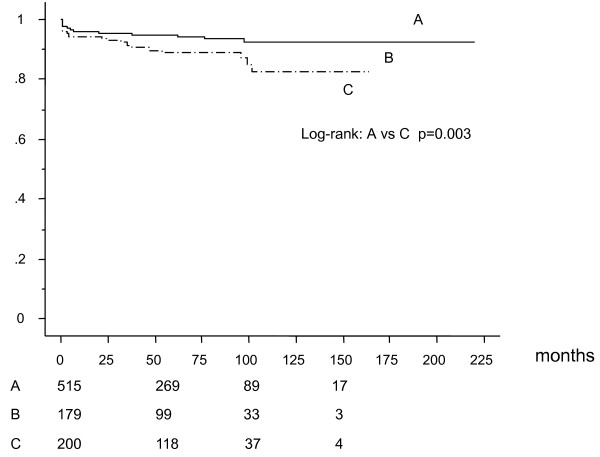
**Freedom from cardiac death**.

**Figure 3:**
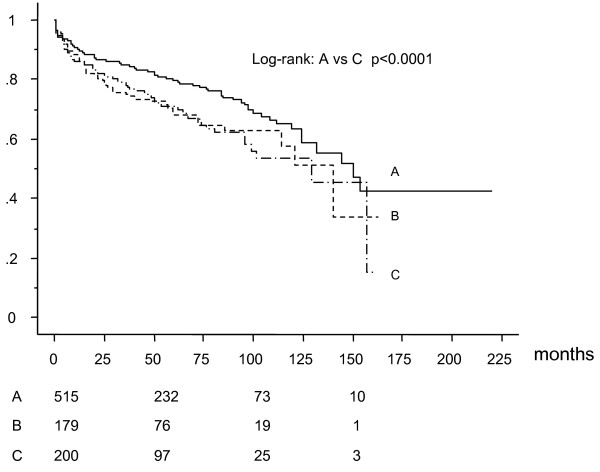
**Freedom from cardiac event**.

Five-hundred fifteen patients underwent CABG with having had no previous PCI (group A), 179 patients with single previous PCI (Group B), and 200 patients with multiple previous PCI (2-15 times, mean 3.6 ± 2.3 times) (group C) before CABG. Table 1 showed preoperative patients characteristics. Preoperative left ventricular ejection fraction was significantly higher in group A (group A;58 ± 13%, group B;54 ± 12%, and group C;54 ± 12%). Table 2 shows angiographic and operative characteristics. There was no significant difference in the extent of coronary artery disease and use of off-pump CABG (OPCAB) technique among the groups. Patients in group C had significantly less bypass grafts than group A and B (group A:3.3 ± 1.0, group B 3.4 ± 0.9, group C 3.1 ± 1.0). Although there was no statistically significant difference among the groups, in-hospital mortality in group C was higher than that in group A and B (group A:1.6%, group B:1.1%, group C:3.5%, p = 0.16). Among the cardiac deaths in the long-term, 7 patients in group A, 4 patients in group B, and 3 patients in group C died for heart failure, 2 patients in group A, 4 patients in group B, and 5 patients in group C died suddenly. One patient in group A, no patient in group B, and 3 patients died for acute myocardial infarction. Survival analysis by Kaplan-Meier method (mean follow-up: 58 ± 43 months) revealed that all-cause death free rate (Figure 1) and cardiac death free rate (Figure 2) were significantly lower in group C than that in group A and B. Cardiac event free rate (Figure 3) was significantly lower in group C than that in group A. Multivariate analysis revealed that age was an independent risk factor for survival, hemodialysis for survival, cardiac death, and cardiac event, LVEF for survival and cardiac death, number of PCI for cardiac death, and number of arterial grafts for cardiac events (Table 3).

**Table 1:** Preoperative characteristics

	Group A	Group B	Group C	*p*	
Age	66.7 ± 9.1	65.6 ± 8.4	65.3 ± 10.1	NS	
Sex(M/F)	385/130	146/33	149/51	NS	
DM	193(37%)	66(37%)	79(40%)	NS	
HL	241(47%)	75(42%)	89(45%)	NS	
HD	25(4.9)	10(5.6)	18(9.0)	NS	
Creatinine (mg/dl)	1.05 ± 0.53	1.06 ± 0.70	1.08 ± 0.53	NS	
LVEF (%)	58 ± 13	54 ± 12	54 ± 12	0.006	A vs C
NYHA	1.8 ± 0.8	1.8 ± 0.9	2.0 ± 0.7	NS	

**Table 2:** Angiographic and operative characteristics

		Group A	Group B	Group C	*p*
		(n = 515)	(n = 179)	(n = 200)	(A vs C)
Number of PCI		0	1	3.6 ± 2.3	
Extent of coronary lesion					NS
	LMT(%)	294 (57)	108 (60)	103 (52)	
	1VD(%)	4 (0)	3 (2)	5 (3)	
	2VD(%)	35 (7)	11 (6)	24 (12)	
	3VD(%)	161 (31)	57 (32)	68 (34)	
Number of grafts		3.3 ± 1.0	3.4 ± 0.9	3.1 ± 1.0	0.03
Number of arterial grafts		1.9 ± 0.8	1.9 ± 0.8	1.8 ± 0.8	NS
OPCAB		58%	50%	53%	NS

**Table 3:** Multivariate analysis for survival, cardiac death, and cardiac event

	**HR**	**95% CI**	***p***
			
Survival			
Age	1.034	1.000-10.69	0.049
Hemodialysis	7.042	3.049-16.39	< 0.0001
LVEF	0.966	0.948-0.985	0.0004
Cardiac death			
Hemodialysis	6.173	2.088-18.180	0.001
LVEF	0.966	0.939-0.993	0.0143
number of PCI	1.189	1.061-1.332	0.0029
Cardiac event			
Hemodialysis	2.262	1.250-4.098	0.007
Number of arterial grafts	0.729	0.570-0.932	0.012

### Discussion

The present study demonstrated adverse impact of repeated previous PCI on late outcomes of subsequent isolated elective CABG. Patients with a history of repeated PCI had significantly lower survival-rate (all-cause death and cardiac death) after CABG as well as cardiac event free rate. Previous studies reported adverse impact of previous PCI before CABG on early clinical outcomes [5-8]. Thielmann and colleagues reported significantly increased risks for in-hospital mortality and major adverse cardiac events after subsequent CABG in patients with a history of multiple PCI [5,6]. Bonaros and colleagues also demonstrated that patients with prior PCI had higher early mortality, major adverse cardiac event rates, and higher perioperative complication rate [8]. Despite these accumulating evidences showing previous repeated PCI as a risk for early clinical outcomes after subsequent CABG, its pathomechanisms are still unclear.

PCI per se has disadvantages over CABG in terms of long-term clinical outcomes. Hannan and colleagues reported a large scale observational study using New York cardiac registries [4]. In their study, CABG was associated with better survival and lower revascularization rate than with PCI. A meta-analysis using 4 randomized trials by Daemen J and colleagues showed significantly lower cardiac event rates including revascularization rate in CABG [9]. With these backgrounds, one question may arise; why is it that long term clinical outcome after CABG is not equivalent regardless of the subgroups with different number of previous PCI?

In our study, LV function was significantly worse in Group C compared in Group A (Group A;58 ± 13%, Group B;54 ± 12%, and Group C;54 ± 12%). It can be speculated that multiple stenting can cause coronary side-branch obstruction or occlusion, which might compromise collateral blood flow and myocardial injury [10], and it might result in worse LV systolic function in patients with previous repeated PCI than that in patients without it. PCI initiates a sequence of inflammatory reactions, which causes endothelial hyperplasia at the site of stenting [11,12] and this inflammatory reaction might spread beyond the stenting sites and promote diffuse lesion of the coronary artery.

The patients with previous multiple PCI required less number of bypass grafts (group A:3.3 ± 1.0, group B 3.4 ± 0.9, group C 3.1 ± 1.0). This could be explained by some reasons. Firstly, Multiple PCI might promote diffuse coronary artery lesion and it makes bypass grafting more difficult and less effective. Multiple arterial grafting may be a better therapeutic option for such a high risk patient with a history of multiple previous PCI. Gaudino and colleagues reported that the use of arterial grafts in cases which previously developed in-stent stenosis improved the angiographic and clinical results [13]. The present study showed that more arterial grafts were an independent factor for preventing cardiac events and the benefit of multiple arterial grafting will be enhanced in the higher risk condition. Secondly, the coronary artery which has been previously treated by PCI will be left untouched at the subsequent CABG, which will be exposed to risks of subsequent restenosis. It has been reported that graft occlusion rate of CABG is superior to re-stenosis rate of PCI. It is assumable that the prognosis of the coronary artery which was once treated with PCI left untouched at the subsequent CABG might be worse than that of coronary artery which would not have been treated with PCI and would have been bypassed with subsequent CABG. Hence, it could be speculated that multiple previous PCI would deteriorate the potential CABG target vessels, which may lead to less number of graft vessels with worse long term survival.

There are limitations in the present study related to its design. The present study was nonrandomized and retrospective study. Although the multivariate analysis showed previous repeated PCI as an independent risk for subsequent CABG, it also could be speculated that the worse clinical outcomes in patients with previous repeated PCI was attributed to the patient's backgrounds of higher coronary risks. The mechanisms were not clarified in the present study. Furthermore, the sample size was limited. More patients need to be studied to confirm the current results.

## Conclusions

Repeated PCI increases risk for long-term prognosis of subsequent CABG.

## Competing interests

The authors declare that they have no competing interests.

## Authors' contributions

GS carried out the acquisition of the data and drafted the manuscript. TS participated in the statistical analysis and interpretation of the data. TK participated in the study design and coordination. All authors read and approved the final manuscript.
